# Supplementary material for: Mid-infrared chemical imaging of intracellular tau fibrils using fluorescence-guided computational photothermal microscopy
Source: Light Sci Appl. 2023 Jun 15;12:147. doi: 10.1038/s41377-023-01191-6 (PMC10272128; doi:10.1038/s41377-023-01191-6)
Supplement: Supplementary file 1 — Supplementary Information [file 41377_2023_1191_MOESM1_ESM.docx]

**Supplementary information for**

**Mid-infrared Chemical Imaging of Intracellular Tau Fibrils using Fluorescence-guided Computational Photothermal Microscopy**

Jian Zhao^1, 2, *^, Lulu Jiang^3^, Alex Matlock^1,4^, Yihong Xu^5^, Jiabei Zhu^1^, Hongbo Zhu^6^, Lei Tian^1,7^, Benjamin Wolozin^3^, and Ji-Xin Cheng^1, 5, 7, 8, *^

^1^Department of Electrical and Computer Engineering, Boston University, Boston, MA 02215, USA

^2^The Picower Institute for Learning and Memory, Massachusetts Institute of Technology, Cambridge, Massachusetts 02142, USA

^3^Department of Pharmacology and Experimental Therapeutics, Boston University School of Medicine, Boston, MA 02118, USA

^4^Current affiliation: Department of Mechanical Engineering, Massachusetts Institute of Technology, Cambridge, Massachusetts 02142, USA

^5^Department of Physics, Boston University, Boston, MA 02215, USA

^6^State Key Laboratory of Luminescence and Applications, Changchun Institute of Optics, Fine Mechanics and Physics, Chinese Academy of Sciences, Changchun 130033, China

^7^Department of Biomedical Engineering, Boston University, Boston, MA 02215, USA

^8^Photonics Center, Boston University, Boston, MA 02215, USA

*Corresponding authors

*E-mail: [jxcheng@bu.edu](mailto:jxcheng@bu.edu) (J.X.C.), jianzhao@knights.ucf.edu (J. Z.)

**Extended data for 3D bond-selective cell imaging**


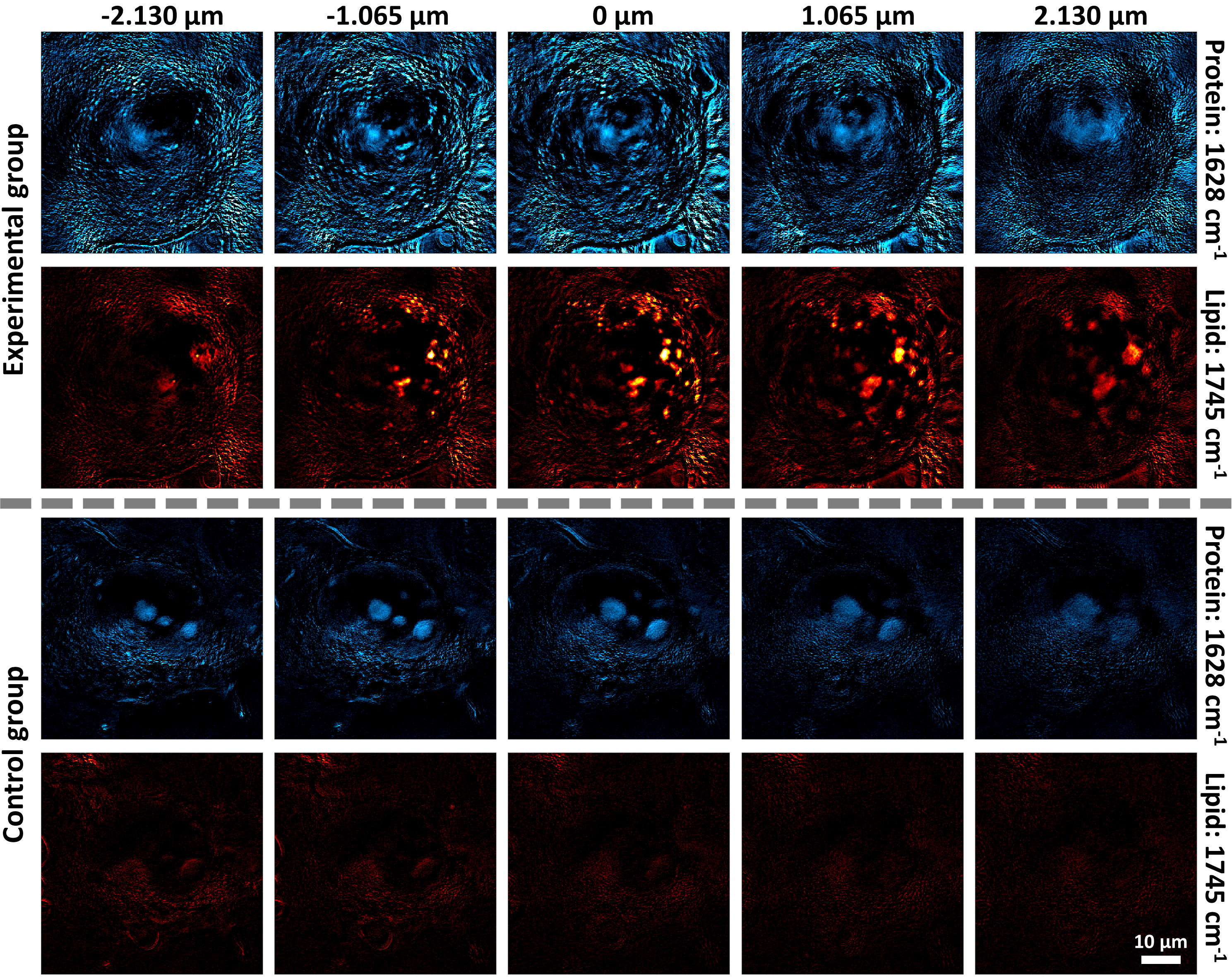


**Fig. S1. Depth-resolved FBS-IDT chemical imaging on human epithelial cells (Tau RD P301S FRET Biosensor cells).** These are the extended data for imaging results shown in Fig. 3 in the main manuscript.

**Extended data for depth-resolved mid-IR fingerprint spectra**

**
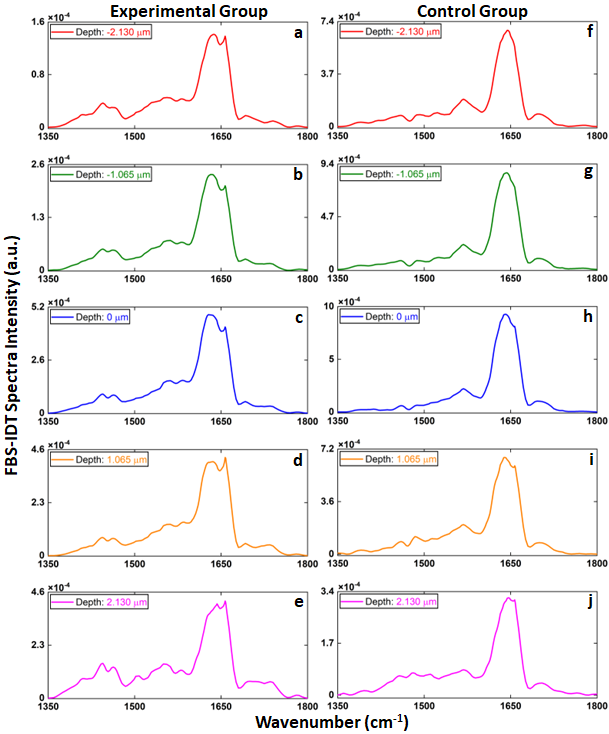
**

**Fig. S2. Depth-resolved mid-IR fingerprint spectra.** **a-e** Depth-resolved mid-IR spectra extracted from the areas shown in Fig. 4c in the main manuscript. **f-j** Depth-resolved mid-IR spectra extracted from the areas shown in Fig. 4f in the main manuscript. The axial depth value of each spectrum is shown in the top left of each plot.


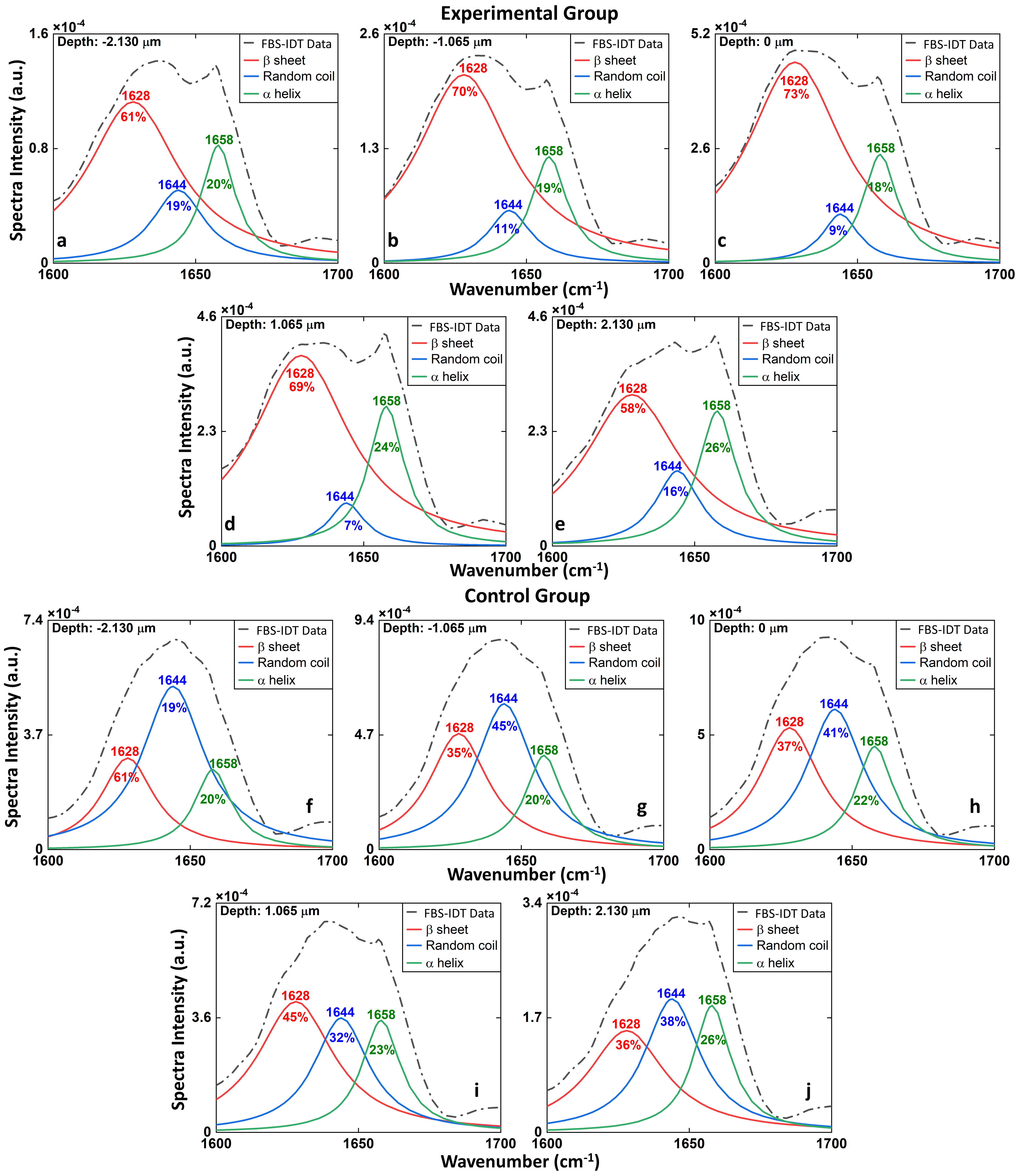


**Fig. S3. Protein secondary structure analyses.** **a-e** Protein secondary structure analyses based on amide I bands for the cell with tau fibrils in the experimental group. **f-j** Protein secondary structure analyses based on amide I bands for the cell without tau fibrils in the control group. The amide I band spectra are extracted from the mid-IR fingerprint spectra shown in Fig. S2. Three main protein secondary structures, the α helix, β sheet, and random coil, are quantified using the deconvolution method. The percentage and the peak positions for each secondary structure are indicated in each plot.

**Timing scheme**

**
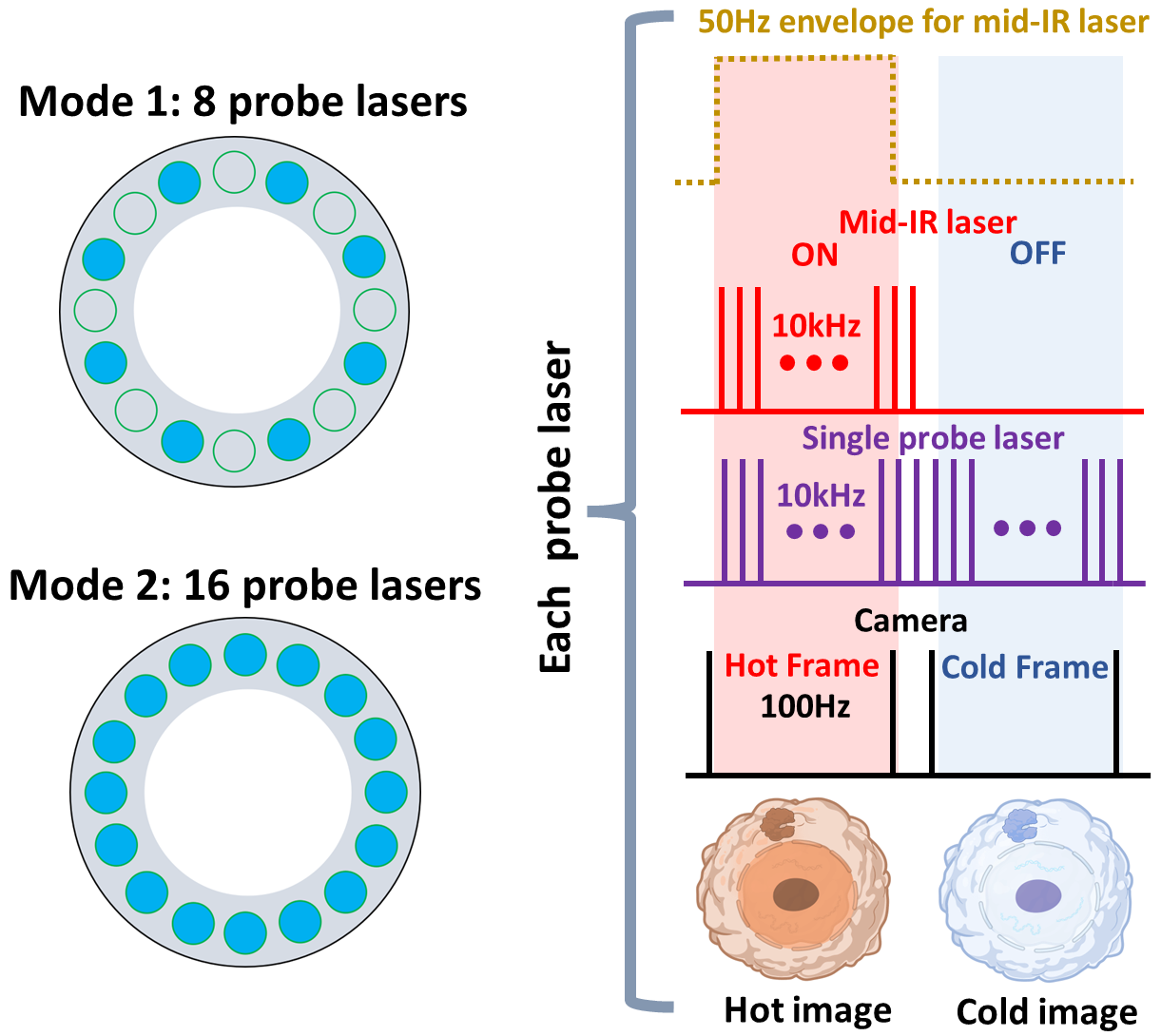
**

**Fig. S4. Timing scheme.** For each probe laser, it runs continuously at 10 kHz with a pulse duration of 1 µs. The mid-IR laser is modulated at the same 10 kHz with the same pulse duration. Each probe laser pulse is synchronized with each mid-IR laser pulse with a ~0.5 µs temporal delay. The camera operates at 100 Hz and is synchronized with the probe laser and the mid-IR laser. To record one “Hot” and one “Cold” image sequentially per probe laser, an additional 50 Hz on/off duty-cycle modulation is imposed on the 10 kHz mid-IR laser pulse train to generate “Hot” and “Cold” states. The above process is repeated for all the lasers in the ring system. Two operation modes are available: 8 probe lasers and 16 probe lasers. Cell icons are created and adapted from^1^.

**3D bond-selective imaging of an artificial phantom**


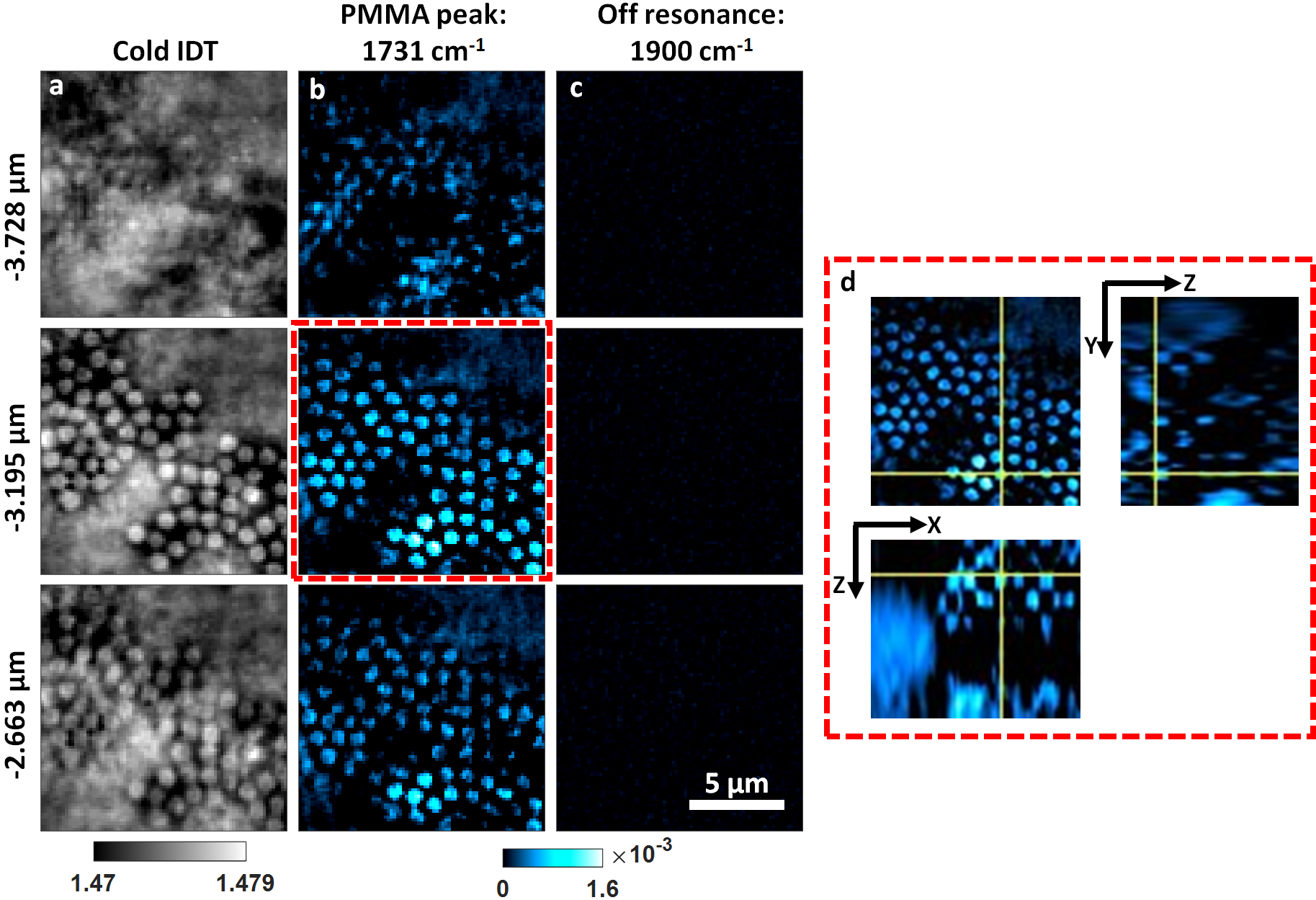


**Fig. S5. Chemical imaging on polymethyl methacrylate (PMMA) beads in soybean oil.** **a** Depth-resolved cold imaging results. **b** Chemical imaging results under PMMA’s absorption peak at 1731 cm^-1^. **c** Chemcial imaging results under off-resonance non-absorptive peak at 1900 cm^-1^. The scale bar in “**c**” is applicable to “**a**-**c**”. **d** Orthogonal view of the red dash-line square area in “**b**”.

We validated the imaging performance of FBS-IDT by performing chemical imaging on an artificial phantom consisting of PMMA beads embedded in soybean oil. The beads and the oil were sandwiched between two pieces of 0.2 mm-thick Raman-grade CaF_2_ glasses. In **Fig. S4**, we demonstrated the depth-resolved chemical imaging results of PMMA beads without or under different mid-IR illumination conditions, as well as the orthogonal view of the sample reconstructions. The FBS-IDT method generally recovers the image of the sample with high fidelity.

**Supplementary movies**

Movie 1. 3D rendering of the chemical imaging on the experimental group cell

Movie 2. 3D rendering of the chemical imaging on the control group cell

Movie 3. 3D rendering of tau fibrils’ β sheet structure

Supplementary information accompanies the manuscript on the Light: Science & Applications website (http://www.nature.com/lsa).

**Reference**

1. BioRender.com.). <https://app.biorender.com/biorender-templates> (2022).
